# Editorial: Applications of Modern Genetics and Genomic Technologies to Enhance Aquaculture Breeding

**DOI:** 10.3389/fgene.2022.898857

**Published:** 2022-04-05

**Authors:** Nguyen Hong Nguyen, Anna K. Sonesson, Ross D. Houston, Hooman Moghadam

**Affiliations:** ^1^ School of Science, Technology and Engineering, University of the Sunshine Coast, Maroochydore, QLD, Australia; ^2^ Norwegian Institute of Food, Fisheries and Aquaculture Research (Nofima), Tromsø, Norway; ^3^ Benchmark Genetics, Edinburgh Technopole, Edinburgh, United Kingdom; ^4^ SalmoBreed AS, Bergen, Norway

**Keywords:** aquaculture genomics, genetics, selective breeding, genetic improvement and genetic lines, substainability

Aquaculture is a critical component of global food security, providing over half of the world’s seafood supply, with projections for significant expansion in the coming decades to meet the increasing demands for animal protein production associated with human population growth. Selective breeding has offered substantial opportunities to enhance production efficiency and help tackle barriers such as diseases *via* cumulative and permanent genetic improvement of farmed strains. With the recent advance in high throughput genome sequencing technologies, coupled with better computational approaches, a large amount of “omics” data have been generated to understand the factors underlying biology and genetic architecture of complex traits and hence, can accelerate genetic gain in aquaculture species. This Research Topic attempted to present and discuss innovative approaches and recent advances in the field of genetics and “omics” to provide solutions to enhance aquaculture breeding. The book included 30 articles (three review and 27 research articles), which are grouped in 15 different themes or topics (Table 1). Main findings and their implications in the context of genetic improvement programs for aquaculture species are summarised as follows:

**TABLE 1:** Main research themes included in the Special Issue.

No	Theme/Topic	Species	Common Name	Main Findings	References
1	Control of reproduction	*Holothuria leucospilota*	Black sea cucumber	RGP to induce spawning	Chieu et al.
2	Genetic diversity	*Labeo rohita*	Rohu carp	9,157 SNPs for sibship analysis	Hamilton et al.
			Yellow Perch	New sets of DNA markers	Guo et al.
3	Quantitative genetic basis and genomic architecture of commercial traits	*Liptopenaeid vannamei*	Pacific whiteleg shrimp	Heritable genetic variation to select for WSSV	Trang et al.
			Turbot	Heritable genetic components for resistance, resilience, tolerance	Saura et al.
4	Genetic improvement programs	*Lates calcarifer*	Asian seabass	Gains for growth and survival	Khang et al.
		*Oncorhynchus mykiss*	Rainbow trout	Gain for fillet yield	Vandeputte et al.
		*Oncorhynchus tshawytscha Perna canaliculus*	King salmon Green mussel	New genomics resources for selection	Symonds et al.
5	New traits	*Fish species*	Fish	Selection for protein to lipid or RFI	Knap and Kause
		*Cyprinus carpio*	Common carp	Meat yield	Prchal et al.
6	Genetic assessment	*Coho salmon*	Coho salmon	Population size using SNPs	Barría et al.
7	Whole genome sequencing and marker development	*Oncorhynchus mykiss*	Rainbow trout	30 million SNPs	Gao et al.
		*Haliotos rubra and H.laevigata*	Blacklip & Greenlip Abalone	Parentage and hybrid grouping	Kijas et al.
8	Linkage maps	*Oreochromis niloticus*	Nile tilapia	50k SNPs	Joshi et al.
9	Functional genomics	*Seriola lalandi*	Yellowtail kingfish	First high-density map	Nguyen et al.
		*Oncorhynchus mykiss*	Rainbow trout	49 gene clusters with growth	Salem et al.
10	Genomic prediction (selection)	*Cyprinus carpio*	Common carp	18% better than pedigree	Palaiokostas et al.
		*Crassostrea gigas*	Pacific oysters	25–30% better than pedigree	Gutierrez et al.
		*Salmo salar*	Atlantic salmon	Accuracy for AGD resistance ∼0.50	Boison et al.
		*Penaeids and Molluscs*	Shrimp and Pearl oysters	Genomic selection for Pearl traits	Zenger et al.
11	Marker-Assisted or genome-based Selection	*Oncorhynchus mykiss*	Rainbow trout	Accuracy of MAS ∼0.5	Liu et al.
		*Lates calcarifer*	Asian seabass	Genome assembly, genes and functions and selection	Orbán et al.
12	Genome assembly	*Pargrus major*	Red sea bream	Frist draft genome assembly	Shin et al.
		*Haliotos rubra*	Blacklip Abalone	High quality genome	Gan et al.
13	Understanding immune response	*Coregonus maraena*	Whitefish	Genes for heat stress	Rebl et al.
		*Salmo salar*	Atlantic salmon	Genes for resistance	Mohamed et al.
		Sparidae	Sea bream	Sex-specific genes	Tsakogiannis et al.
14	Genome/gene editing	*Acipenser ruthenus*	Sterlet	Reduced maturation time	Chen et al.
15	Genomic tools	*Molluscs*	Molluscs	Genetics and omics methods in mollusc	Hollenback and Jonhston
		*Penaeids*	Shrimp	State of omics in penaeids	Guppy et al.

**Theme 1** (Reproduction biology): Control of reproduction is the first step before initiating captive breeding programs for any aquaculture species. In efforts to develop a new genetic and stock enhancement program for black sea cucumber *Holothuria scabra*, Chieu et al. identified the neurohormone relaxin-like gonad-stimulating peptide (RGP) and a recombinant RGP was produced to induce spawning in this species. Spawned oocytes were fertilized successfully, larvae settled and developed into juveniles. These results demonstrated the roles of neuropeptides involved in sea cucumber reproduction that can provide a new artificial breeding approach in sea cucumber aquaculture.

**Theme 2** (Genetic diversity of founder stocks): Assessing genetic diversity of founder stocks is another important step to form a base population with large genetic variability to ensure long term response to selection. In this regard, Hamilton et al. used a new generation of DNA markers (i.e., single nucleotide polymorphism, SNPs) to analyse sibship relationship among founder stocks of rohu carp *Labeo rohita*. Guo et al. also attempted to develop new sets of SNPs to examine genetic diversity of yellow perch *Perca favescens*. Results from these studies demonstrated that DNA markers can assist the identification and recruitment of genetically diverse stocks for selective breeding programs.

**Theme 3** (Genetic basis of production traits): Once the base population is established, it is necessary to know genetic parameters for key traits of interest, to guide the breeding goal and response to selection. In Pacific whiteleg shrimp *Liptopaneus vannamei*, Trang et al. analysed a pedigree comprising 15,000 individuals and reported there are heritable genetic variation for disease resistance to white sport syndrome virus, WSSV (mean heritability for WSSV across statistical models = 0.12). There were also strong and positive genetic correlations of WSSV resistance between different infection periods. The genetic associations between WSSV resistance and growth traits, albeit negative, were not significant. Improving the definition and recording of target traits is also important. In Turbot *Philasterides dicentrarchi*, Saura et al. used genotype data (18,125 SNPs) derived from genotyping by sequencing platforms to understand genomic architecture of disease resistance, resilience and tolerance. The SNP heritabilities for these traits were moderate to high, indicating that selection to improve disease resistance can be effective in aquaculture species.

**Theme 4** (Designing breeding programs): Genetic selection has significantly improved productivity and quality of several aquaculture species. In this book, Khang et al. reported an 8-years breeding program for improved growth and survival in a marine finfish, Asian seabass *Lates calcarifer*. In addition, they used the data collected over 8 years to estimate genetic parameters for body traits and survival during growth trajectory and investigated the effect of genotype by culture system (tank vs sea cage) interaction to optimise the design and conduct of the breeding program for this species. In addition to growth and survival, Vandeputte et al. demonstrated that selection for carcass weight also increased genetic gain for fillet yield by 1.16% per generation in rainbow trout *Oncorhynchus mykiss*. The selection programs in both Asian seabass and rainbow trout have based on pedigree and phenotype information. In breeding programs for two main species farmed in New Zealand: Chinook (king) salmon *Oncorhynchus tshawytscha* and green shell mussel *Perna canaliculus*, new high through-put sequencing platforms (i.e., genotyping by sequencing) are being used to generate genomic resources to incorporate into breeding selection schemes for these species (Symonds et al.).

**Theme 5** (New traits of commercial importance): Broadening the breeding objectives in genetic programs for aquaculture species is essential to maximise productivity and revenue of aquaculture enterprises. In this regard, Knap and Kause proposed two new measures to improve efficiency of food utilisation, i.e., protein to lipid ratio and residual feed intake. Prchal et al. also attempted recording morphological measurements by 2D digitalization and ultrasound imagery to predict fillet yield in common carp *Cyprinus carpio*. While the results of both studies are encouraging, benefit-cost analysis of these technologies should be evaluated in practical breeding programs in fish species, because recording protein (lipid) content or individual feed intake as well as carcass traits using ultrasound tomography are laborious and expensive.

**Theme 6** (Monitoring of breeding programs): Assessment of genetic programs should be frequently conducted to monitor the progress of the selected population and to refine breeding technologies. Barría et al. used a 200K high-density SNP array to estimate population size of 43 (10 generations ago) to 325 (139 generations ago) in Coho salmon *Oncorhynchus kisutch*. In closed breeding nucleus, the population size should be at least 50 to maintain an acceptable level of inbreeding below 1% per generation. The effective population size is also related to genetic diversity and adaptation and evolution of captive populations.

**Theme 7** (Next generation sequencing and marker development): The advent of next generation sequencing enables the simultaneous genotyping of hundreds of thousands, or millions of DNA markers used for genetic and genomic studies in various aquaculture species. Well-designed SNP panels can support breeding programme design and operation in multiple ways. One example is, through whole genome resequencing, approximately 30 million SNPs were identified on the rainbow trout genome and functional annotation based on the genome position of each SNP, which provide an important source to design new high-density SNP arrays (Gao et al.). This approach was used to develop a SNP genotyping tool for parentage assignment and grouping of hybrids animals from their parents in blacklip *Haliotos rubra* and greenlip *H. laevigata* abalone (Kijas et al.).

**Theme 8** (Linkage maps and QTL analysis): With the abundance of SNPs markers, two studies in this Special Issue attempted to develop genetic linkage maps for two important fish species. First, Joshi et al. developed a 58k SNP-array and a high-density linkage map for Nile tilapia *Oreochromis niloticus*. The linkage map was integrated with the *O niloticus* genome to produce an integrated physical and genetic linkage map comprising 40,186 SNPs distributed across 22 linkage groups. Second, Nguyen et al. also developed a first high-density linkage map involving 4,000 SNPs distributed across 24 linkage groups for yellowtail kingfish *Seriola lalandi*. Results from these results revealed a general pattern of recombination across the genome was notably different between the sexes, with female recombination rates being higher across many regions of the genome.

**Theme 9** (Functional genomic analysis): In addition to polymorphic DNA markers generated from high through-put genome sequencing, Salem et al. used a 50k transcribed gene SNP-chip derived from RNA-seq data and identified several QTL regions explaining up to 28.4% of the additive genetic variance for muscle yield in rainbow trout *O. mykiss*, with 46 SNPs clustered into 23 annotated genes on chromosome 14 or 49 SNPs clustered into 16 annotated genes on chromosome 16. Examples of the annotated genes in these QTL regions are fibroblast growth factor-binding protein-1(FGFBP1) which had a single nonsynonymous SNP found in a window that explained 12.24% of the additive genetic variance or the cysteine/serine-rich nuclear protein 2 (CSRNP2) or the citrate synthase (CS). However, the CS assay showed no association between any of the SNP genotypes and CS activity. The author suggested an important role of mitochondrial functions to muscle growth as reported in earlier studies in human. In yellowtail kingfish, Nguyen et al. used 13,898 SNPs generated by genotyping by sequencing platforms and reported that there were six SNPs significantly associated with growth. The significant SNPs are located in (near) genes with known functions linked with signal transduction, cytoskeleton, membrane channels and ions transport. However, these SNPs explained only a limited proportion of the additive genetic variance, only 3.58%. Results from these studies suggest that growth and quantitative traits are involved with complex processes controlled by multiple genes and gene families. Functional characterization of the significant markers deserves further study to elucidate their biological functions underlying growth and complex traits in kingfish as well as other aquaculture species.

**Theme 10** (Genomic selection): Genomic selection is of practical significance in aquaculture species especially for traits that are difficult to measure (e.g., meat quality, disease resistance) or for those with low heritability (e.g., fitness-related traits). Palaiokostas et al. showed that genomic prediction of breeding values using 12,311 SNPs derived from Restricted site associated DNA (RAD) sequencing outperformed pedigree-based prediction by 18% for early growth in juvenile common carp *C. carpio*. Similarly, the superiority in the prediction accuracy for growth-related traits were from 25 to 30% in Pacific oyster when a medium density SNP array (23k SNPs) was used (Gutierrez et al.). The prediction accuracy for resilience to *Philasterides dicentrarchi*, a parasite causing scuticociliatosis in Turbot was 0.46, which was also higher than the estimate obtained from the pedigree, 0.41 (Saura et al.). The genomic prediction accuracy varies from 0.16 to 0.83 (median = 0.60) for various disease survival traits in aquatic animal species, which open opportunities to select for quality traits (e.g., lustre, complexion, shape, size and colour) in highly economic value species, such as Pearl oyster *Pinctada maxima* (Zenger et al.).

**Theme 11** (Marker- or gene-assisted selection): In addition to genomic selection, which is based on genome-wide marker information, two studies in this Issue attempted to identify genes (variants) with moderate to large effects to enable gene- or marker-assisted selection (MAS) in rainbow trout *O. mykiss* and Atlantic salmon *Salmo salar*. Liu et al. incorporated 36 SNPs (clustered mainly on chromosomes 3, 5, and 8 of rainbow trout) into the selection program for Bacterial cold water disease (BCWD), caused by *Flavobacterium psychrophilum* in rainbow trout, and demonstrated that accuracy of MAS was around 0.50, which was equal or greater than the accuracy of the conventional family-based selection in the same generation. Another successful example of MAS is for IPN (Infectious Pancreatic Necrosis) resistance in Atlantic salmon in Norway and the United Kingdom (reviewed by Orbán et al. in this Issue).

**Theme 12** (Reference genome assembly): An essential genomic tool is a sequenced and assembled genome for a species of interest. Shin et al. constructed the first draft genome, with a size of 829.3 Mb, using the 90X PacBio sequence, for red sea bream of Sparidae family. Additionally, Gan et al. used Nanopore long reads (∼30x coverage) and applied hybrid meta-assembly approach to assemble the first high-quality genome assembly for Blacklip abalone. The authors also annotated the genome and explored the genome for heat-shock proteins (HSPs), a highly conserved protein family whose synthesis has been previously linked to thermal stress in *Haliotis* species. To date, the assembled teleost genomes is around a hundred, but it will likely double in coming years through Vertebrate Genome Project. Fully assembled and annotated genomes will assist future selective breeding and fundamental genomic and evolutionary studies for important aquaculture species.

**Theme 13** (Understanding immune response): Boison et al. performed whole transcriptome profiling of normal and infected fish to understand molecular mechanisms underlying genetic resistance to Amoebic gill disease (AGD), caused by the infection of the protozoan *Neoparamoeba perurans*, a primary gill disease of farmed Atlantic salmon (*Salmo salar*) around the globe. The study identified three main QTL regions on chromosomes 4, 9 and 13 linked with AGD resistance in this species. The QTL region on chromosomes 4 harbours members of the cadherin gene family, the region on chromosome 9 associated with another member of this gene, i.e., protocadherin Fat 4, and the region on chromosome 9 covering interleukin-18 (IL18) binding protein, a gene playing a crucial role in inhibiting proinflammatory effect of cytokine IL18. In Maraena whitefish (*Coregonus maraena*), Rebl et al. performed microarray-based transcriptome analysis of three main tissues (liver, spleen and kidney) and detected 112 differentially expressed genes related to thermal stress induced by gradual or acute temperature rise in these tissues. Examples of genes that are upregulated after heat shock include HSP40, HSP70 and HSP90; their functional homologs, co-chaperons and stress-signal transducers are conserved across different species. Furthermore, Tsakogiannis et al. reported male and female-specific genes and their pathways that can improve efforts to control phenotypic sex in gilthead seabream.

**Theme 14** (Genome editing): Genome (gene) editing has some potential applications to enhance aquaculture breeding, albeit there remains some controversy in the potential use of this technology. In this Issue, Chen et al. established gene transfer and gene editing techniques to shorten sexual maturation time in Sturgeon species, which normally range from 6 to 12 years in males and 10—18 years in females. The authors reported that the clustered regularly interspaced short palindromic repeats (CRISPR)/Cas9 gave better results than transcription activator-like effector nucleases (TALENs) in terms of embryo survival rate. The study also successfully edited *ntl* (no tail) gene and produced sturgeon fries with phenotypes similar to those observed after *ntl* knockdown using morpholino technology in zebrafish.

**Theme 15** (Genomic tools): Hollenback and Jonhston and Guppy et al. presented the state of current “omic” technologies used in genetic selection programs for mollusc and Penaeids, respectively. The reviews covered the current literature in genomics, transcriptomics and to a lesser extent, proteomics as well as genetic methods such as linkage maps, QTL analysis, genome-wide association studies, genomic prediction and functional genomics. However, the “genomic tools” lack important components of emerging areas of research, namely metagenomics, epigenomics etc, which display potential applications to enhance aquaculture breeding, especially for traits which are difficult to measure (e.g., disease resistance) or for those which are largely influenced by genotype × environment interaction. These areas of research merits further research to optimise genetic improvement programs for aquatic animal species.

In summary, this Special Issue (book) presented a systematic and integrated approach for the design and conduct of genetic enhancement programs, from reproduction biology to assessment of genetic diversity to recruit founder stocks to form base populations, successful implementation of selective breeding programs, as well as the application of gene- (marker-) assisted selection and the incorporation of new “omics” resources to accelerate genetic progress made in selective breeding programs for aquaculture species. To date, genetic improvements in aquaculture species have been based mainly on the application of quantitative genetics theories and conventional selective breeding approach. Genome-based selection has emerged as a new paradigm in genetic enhancement programs for both terrestrial and aquatic animal species. In coming years, we are expecting to see more the application of other omics resources (e.g., proteomics, metabolomics) in practical selective breeding programs for aquaculture species of economic importance.

Finally, we would like to thank all the authors, reviewers and editors who have made this Research Topic a success.

